# Satisfaction of healthcare workers and patients regarding telehealth service in a hospital in Peru

**DOI:** 10.17843/rpmesp.2022.394.11287

**Published:** 2022-12-30

**Authors:** Fernanda Barriga-Chambi, Fabricio Ccami-Bernal, Alberto Luciano Alarcon-Casazuela, Julissa Copa-Uscamayta, Jeremy Yauri-Mamani, Brenda Oporto-Arenas, Cender Udai Quispe-Juli

**Affiliations:** 1 Universidad Nacional de San Agustín de Arequipa, Arequipa, Peru Universidad Nacional de San Agustín Universidad Nacional de San Agustín de Arequipa Arequipa Peru; 2 Sociedad Científica de Estudiantes de Medicina Agustinos (SOCIEMA), Arequipa, Peru. Sociedad Científica de Estudiantes de Medicina Agustinos Arequipa Peru; 3 Universidad Peruana Cayetano Heredia, Lima, Peru. Universidad Peruana Cayetano Heredia Universidad Peruana Cayetano Heredia Lima Peru

**Keywords:** Telemedicine, Remote Consultation, COVID-19, Patient Satisfaction, Health Personnel

## Abstract

**Objectives.:**

To evaluate the level of satisfaction of healthcare workers and patients with the telehealth service of the Hospital III Regional Honorio Delgado (HRHD), as well as the maturity level of the telehealth service implementation.

**Materials and methods.:**

Cross-sectional, observational study conducted from October to December 2021. The satisfaction of healthcare workers and patients was assessed with the Glaser et al. survey and the Telemedicine Satisfaction Questionnaire (TSQ), respectively. The level of service maturity was assessed using the Pan American Health Organization's instrument for measuring the maturity level of healthcare institutions implementing telemedicine service.

**Results.:**

A total of 129 responses were obtained from healthcare workers. Non-physician professionals' satisfaction with the telehealth service was higher than that of physicians (72.5% vs. 18.3%). Of 377 patients, 77.6% stated they were satisfied with the service. Regarding the maturity level, the HRHD telemedicine service had 32% of items in null status, 40.8% in started, 25.2% in advanced, and 2% in ready conditions.

**Conclusions.:**

Physician satisfaction was lower than that of other health professionals. Patients had a moderate-high satisfaction. The maturity level of telehealth implementation in HRHD was oriented towards a null or initiated level. Decision-makers need to consider user satisfaction for the telehealth implementation and the follow-up.

## INTRODUCTION

The COVID-19 pandemic has challenged the face-to-face care capacity of healthcare systems, forcing them to redesign and/or implement other methods of patient care via telehealth ^1^. According to WHO, telehealth is the delivery of health services remotely using Information and Communication Technologies (ICT) for the diagnosis, treatment, and prevention of diseases ^2^. It has advantages such as better access, optimization of resources, minimization of costs, lower risk of contagion, among others ^3^. On the other hand, there are concerns about regulatory gaps, ICT infrastructure, personnel training, digital divide, security, and privacy, among others, that constitute barriers to its implementation ^4^. The implementation and sustainability of these services depend to a large extent on the perception and satisfaction of patients and healthcare personnel (HCP), since they are the main source of information on whether medical care is provided correctly and whether it meets their expectations ^5^. 

Patient satisfaction is influenced by several factors, such as service delivery (availability of a specialty, frequency of care, obtaining information, outpatient, or inpatient treatment), the doctor-patient relationship and technological factors (access to the system, absence of technical problems) ^1^. Previous studies found high patient satisfaction, ranging from 68% to 100% ^6^^,^^7^. Few studies have evaluated the satisfaction of health personnel, (satisfaction reported between 64%-81%) ^8^. It has been reported that patient satisfaction with the telehealth service through telephone calls was higher than that of health personnel ^9^. Despite the development of telehealth initiatives during the COVID-19 pandemic, few studies in Latin America and the Caribbean (LAC) have evaluated patient and/or health personnel satisfaction ^10^; there are no studies published in indexed Peruvian journals.

Few countries in LAC have well-established telehealth policies and regulations, therefore knowing about the satisfaction of patients and health personnel - as an element of feedback - would allow redirecting regulations in order to improve healthcare via telehealth ^11^. The aim of the present study was to evaluate the level of satisfaction of health personnel and patients who received care by the telehealth service at the Hospital Regional III Honorio Delgado (HRHD) in Arequipa, Peru. The secondary objective was to evaluate the level of maturity of telehealth implementation at the HRHD.

**KEY MESSAGES**

Motivation for the study: it is important to understand the satisfaction of users regarding the rapid implementation and increasing use of telehealth due to the COVID-19 pandemic. There are few studies in Latin America and none in Peru.

Main findings: satisfaction of non-medical health personnel is moderately high, contrasted with low satisfaction of medical personnel. Patient satisfaction is moderately high. The implementation of telehealth in the Hospital III Regional Honorio Delgado is at a null or initiated level, and could reflect the situation in several centers of the National Telehealth Network.

Implications: understanding the level of user satisfaction will allow the standardization of action plans, as well as improving the implementation, refinement, and follow-up of telehealth services in order to improve the quality of health care being provided.

## MATERIALS AND METHODS

### Study design and location

This was a cross-sectional observational study, with a descriptive approach. It was conducted at the HRHD of Arequipa, which belongs to the National Telehealth Network. The telehealth service was quickly implemented in this hospital during August 2020 due to the COVID-19 pandemic. The HRHD uses the "Teleatiendo" platform to manage appointments, record care data and other related processes. The HRHD has been recognized twice as one of the healthcare institutions with the highest number of "Teleatiendo" appointments nationwide. It is important to point out that all the services are provided via telephone calls. The earliest record found on the platform dates back to May 2021 with 4951 visits, reaching a maximum of 10,714 in January 2022 (supplementary material, Annex 1).

### Population and sample

The study population consisted of HRHD HCP who provided telehealth care during the period 2020-2021 and patients who received care by the HRHD telehealth service from October 15 to December 15, 2021. The participation of the entire HCP was sought. We used stratified random sampling of patients. To calculate the sample size, the total number of patients registered in the "Teleatiendo" platform during the months of August and September (20,828 patients) was taken as reference. The calculated sample size was 377 patients, with a confidence level of 95% and a percentage frequency of the event of interest of 50%. The strata considered were: telecounseling, telemonitoring and teleconsultation (modalities of care in "Teleatiendo") with a ratio of 28/5/1. The selection of patients was random, weekly, and proportional to the strata during the study time and was carried out until the minimum calculated sample size was reached.

We included the HRHD HCP that carried out telehealth appointments during 2020-2021 and the patients who received care by the HRHD telehealth service of the department of Arequipa who had at least one appointment registered in the "Teleatiendo" platform. HCP with less than six months of telehealth care experience were excluded; patients who denied having received care by the HRHD telehealth service were excluded.

### Variables and instruments

Satisfaction of the HCP was assessed with the Glasser *et al*. survey ^12^, operationalized using a Likert scale. The answer options for the first two questions were: strongly agree, agree, neutral, disagree and strongly disagree; the options for the last three questions were: completely satisfied, satisfied, neutral, dissatisfied, completely dissatisfied. Other variables we considered were: sex, age, profession, time practicing the profession (years), time of telehealth experience (months), telehealth experience prior to the pandemic, frequency of telehealth care (daily, weekly, monthly), perception of competence in the use of telehealth technology (beginner, intermediate, advanced).

Patient satisfaction was assessed with the validated and widely used Telemedicine Satisfaction Questionnaire (TSQ) ^13^, and was operationalized by means of a Likert scale into strongly agree, agree, neutral, disagree and strongly disagree; the last question on overall satisfaction was grouped into two categories: satisfied (strongly agree and agree) and not satisfied (neutral, disagree and strongly disagree). We also collected information on the following sociodemographic variables: health insurance, place of origin (province of Arequipa, other provinces), Internet access, "Teleatiendo" modality (telecounseling, telemonitoring and teleconsultation), attending staff (physician or non-physician, which included nurse, obstetrician, psychologist), specialty of service-providing unit (medicine, surgery, pediatrics, gynecology and obstetrics, and immunizations) and the appointment motive. For the "appointment motive" variable, we reviewed and classified the appointments registered in "Teleatiendo" for each patient according to the main activity carried out during the appointment (counseling, follow-up, or consultation), considering previous appointments and indications. Additionally, at the end of the survey, the HCP and patients were asked a free question about their comments and suggestions about the platform and care.

We used the Pan American Health Organization (PAHO) tool to measure the level of maturity of health institutions for assessing the maturity level of HRHD's teleconsultation system, which was operationalized according to the level of progress in the implementation of telemedicine services as: null (no initiative), started (with progress, but far from what is necessary), advanced (good progress) and ready (functioning at full capacity). This instrument has six dimensions: 1) organizational preparation (bases that identify issues to be resolved before moving forward with telemedicine services), 2) processes (set of operations and functions to be considered), 3) digital environment (necessary technological infrastructure), 4) human resources (institutional capacity in the areas of health services and ICT), 5) regulatory aspects (standards and procedures for the provision of Telemedicine services), 6) specialized knowledge (additional knowledge to benefit effective implementation) ^14^.

### Procedures

Permission from HRHD was requested in order to conduct the study and access the database of the HCP and telehealth user patients during October to December 2021. The surveys were conducted via telephone calls by the researchers and nine previously trained collaborators. The language of the questions was standardized based on a pilot test in order to facilitate the application of the instrument; the terms were also adapted for better understanding.

After being notified about the study by the telehealth service, the HCP were surveyed outside of working hours; in case of no response, a message was left and they were called up to five times at different moments during the day. Patients were surveyed mostly during the morning and evening, in case there was no response, they were called up to two times. The purpose of the study was explained to each respondent in order to obtain verbal consent. To reduce recall bias, patients were surveyed up to two weeks after their care. Epidemiological data and those related to the telehealth appointment were complemented with data from the "Teleatiendo" platform, which were obtained by means of a collection form.

Information on the maturity level was collected during three field visits by two observers (FBC, ALAC), who collected information through observation, dialogue with health and administrative personnel, and verification of evidentiary elements in the telehealth service, according to the PAHO instrument. Questions that were not answered by the previously established techniques were addressed through an interview with the secretary and the head of the HRHD telehealth office. The researchers met and reached consensus on the final score for each indicator.

### Statistical analysis

Data was stored and processed in a Google(r) spreadsheet as well as in a Microsoft Excel 2019(r) file. Data forms with incomplete data were eliminated. Measures of central tendency and dispersion (numerical variables), and absolute and relative frequencies (categorical variables) were calculated. The chi-square test, Fisher's exact test and Mann-Whitney U test were used as appropriate to compare the groups; a value of p<0.05 was considered statistically significant. Statistical analysis was carried out with the STATA 16.0 statistical program.

### Ethical Aspects

Participants were informed about the purpose of the study, then we requested verbal consent to be surveyed and recorded; no incentives were provided. Data were anonymized. This study was guided by the principles of responsible conduct in research and scientific integrity, and was approved by the Institutional Research Ethics Committee of the Avendaño Clinic (code: 017-2021-CIEI), and the Ethics Committee of the HRHD by official letter N°-261-2021 GRA/GRS/GRS/GR-HRHD/DC-OCSI, for authorization. It was also registered in the PRISA platform of the Instituto Nacional de Salud with code EI00000002465.

## RESULTS

We contacted 125 physicians and 173 nonphysician professionals, of whom 60 physicians and 69 nonphysician professionals agreed to participate and responded correctly (nonresponse rate of 52% and 60.1%, respectively). Of the non-medical professionals, 60 (87%) were nurses, 5 (7.2%) obstetricians and 4 (5.8%) psychologists. The proportion of women who were non-medical professionals was higher (100%) compared to the proportion of women who were physicians (58.3%). The average work experience was 24.5 years in physicians and 28.6 years in non-physicians. The proportion of physicians with previous telehealth experience was higher than that of nonphysician professionals (20% vs 1.5%); but the number of nonphysicians working with the telehealth system on a daily basis was higher (50% vs 89.9%) (Table 1).

**Table 1:** Characteristics of health personnel who performed teleconsultations at the Honorio Delgado Regional Hospital of Arequipa (N=129).

Characteristics	Medical personnel n=60 n (%)	Non-medical personnel n=69 n (%)	p-value
Sex			
Women	35 (58.3)	69 (100.0)	<0.001^a^
Men	25 (41.7)	0 (0.0)	
Age group			
Youth and adults (18-59)	36 (60.0)	27 (39.1)	0.018^b^
Elders (≥60)	24 (40.0)	42 (60.9)	
Years of professional experience	24.5 (10.5)	28.55 (13.9)	
Months of telehealth experience	11.93 (4.6)	12.22 (4.5)	
Previous experience in telehealth			
Yes	12 (20.0)	1 (1.5)	0.001^a^
No	48 (80.0)	68 (98.5)	
Frequency of appointments			
Daily	30 (50.0)	62 (89.9)	<0.001^a^
Weekly	27 (45.0)	7 (10.1)	
Monthly	3 (5.0)	0 (0.0)	
Perception of technology use			
Beginner	8 (13.3)	17 (24.6)	0.278^b^
Intermediate	41 (68.3)	41 (59.4)	
Advanced	11 (18.4)	11 (16.0)	

^a^ Fisher's exact test; ^b^ Chi-square

A total of 1,912 patients were contacted; the non-response rate was 80.3%, but this did not alter the calculated sample size (n=377); 76.7% were women and the mean age was 33.8 and 35.5 years for women and men, respectively. Most of the population had high school education. Most had comprehensive health insurance (82.2%) and were from the province of Arequipa (86.7%). The telecounseling modality was the most used (83%). Digital illiteracy (referring to the level of unawareness of new technologies and the need for support in the use of technology by patients) was found in 46.2% of the participants (Table 2).

**Table 2:**
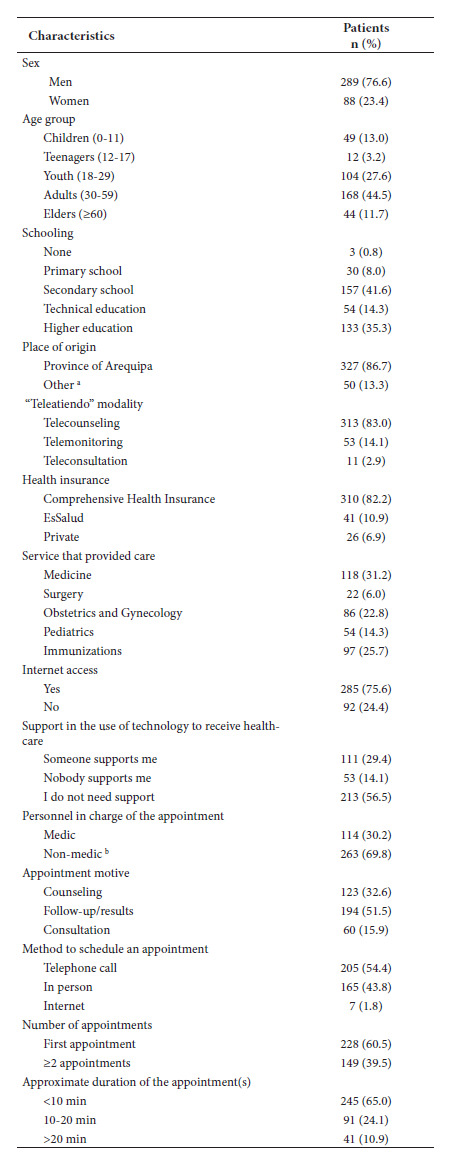
Characteristics of patients attended by the telehealth service of the Honorio Delgado Regional Hospital of Arequipa by telephone call (N=377).

^a^ Other provinces of the Arequipa region other than the province of Arequipa;

^b^ Nurse, obstetrician, psychologist

### Satisfaction of health personnel

The proportion of satisfied and completely satisfied non-physician professionals was significantly higher than that of physicians; 69.6% of non-physician professionals reported being satisfied and completely satisfied with the outcome of the telehealth appointment compared to 38.3% of physicians. Likewise, the level of satisfaction with the "Teleatiendo" platform of non-physician professionals during their last appointment was higher than that of physicians (72.4% vs. 18.3%). The same was true for the perception of the level of satisfaction by their patients in non-physician and physician professionals (87% and 28.3% respectively) (Table 3). The summary of the HCP comments and suggestions can be found in the supplementary material, Annexes 2 and 3.

**Table 3:** Level of satisfaction with the teleconsultation service by healthcare personnel of the telehealth service of the Honorio Delgado Regional Hospital of Arequipa (N=129).

Questions	Strongly agree	Agree	Neutral	Disagree	Strongly disagree	p-value
Having the appointment could change (improve) the patient's prognosis?						
Medical personnel	6 (10.0)	21 (35.0)	18 (30.0)	8 (13.3)	7 (11.7)	0.004^b^
Non-medical personnel ^a^	3 (5.0)	32 (46.4)	10 (14.5)	7 (10.1)	1 (1.5)	
The clinical decision-making process was satisfactorily achieved							
Medical personnel	3 (5.0)	14 (23.3)	17 (28.3)	14 (23.3)	12 (20.0)	0.001^b^
Non-medical personnel ^a^	10 (14.5)	30 (43.5)	20 (29.0)	7 (10.1)	2 (2.9)	
	Completely satisfied	Satisfied	Neutral	Dissatisfied	Completely dissatisfied	
How satisfied are you with the outcome of the appointment?							
Medical personnel	3 (5.0)	20 (33.3)	13 (21.7)	17 (28.3)	7 (11.7)	<0.001^b^
Non-medical personnel ^a^	16 (23.2)	32 (46.4)	17 (24.6)	4 (5.8)	0 (0.0)	
How satisfied were you with the telehealth platform during your last appointment?							
Medical personnel	2 (3.3)	9 (15.0)	18 (30.0)	20 (33.3)	11 (18.3)	<0.001^b^
Non-medical personnel ^a^	15 (21.7)	35 (50.7)	12 (17.4)	5 (7.3)	2 (2.9)	
How do you rate patient satisfaction during the last appointment?							
Medical personnel	5 (8.3)	12 (20.0)	13 (21.7)	19 (31.7)	11 (18.3)	<0.001^b^
Non-medical personnel ^a^	26 (37.7)	34 (49.3)	7 (10.1)	2 (2.9)	0 (0.0)

^a^ Nurse, obstetrician, psychologist; ^b^ Chi-square

### Patient satisfaction

**Figure 1:**
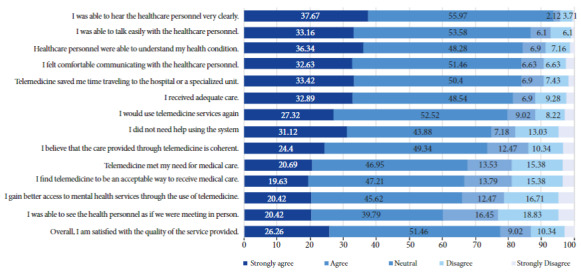
Level of satisfaction with the teleconsultation service by patients attended by the telehealth service of the Honorio Delgado Regional Hospital of Arequipa (N=377).

Of the total participants, 77.7% stated that they strongly agreed or agreed that they were satisfied with the telehealth service. Seven of the thirteen evaluated criteria showed high levels of satisfaction, ranging from 79.8% ("I would use telemedicine services again") to 93.6% ("I could hear the health personnel clearly"). For the remaining six, satisfaction ranged from 60.2% ("I could see the HCP as if we were meeting in person") to 75% ("I did not need help using the system") (Figure 1).

**Figure 2:**
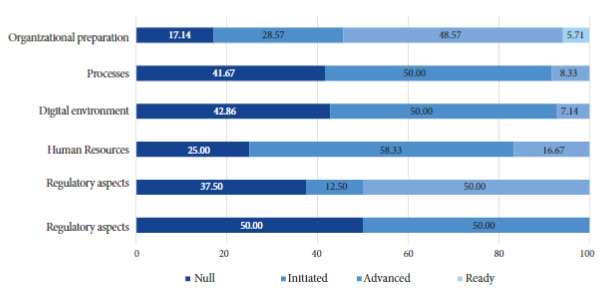
Maturity level of the HRHD telehealth service by category according to the PAHO tool for measuring the maturity level of health institutions for implementing telemedicine services.

Of the satisfied patients, 76.5% were women and 83% used the telecounseling modality. The pediatrics service showed the highest degree of satisfaction compared to other services (87%), followed by gynecology and obstetrics (79.1%). The appointment motive for 54.3% of satisfied patients was "follow-up." A similar level of satisfaction was found among patients attended by medical (76.3%) and non-medical (77.7%) staff. Of the appointment motives, "consultation" had the highest proportion of dissatisfied compared to satisfied (22.2% vs 14%) patients (Table 4). A summary of patient comments and suggestions can be found in the supplementary material, Appendix 4.

**Table 4:**
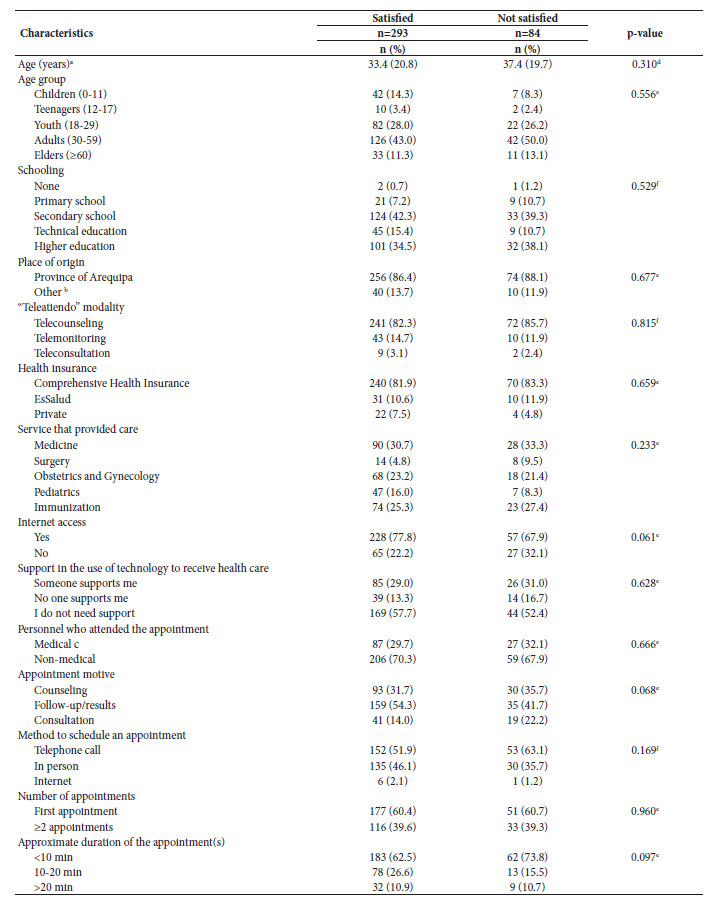
Characteristics of the patients according to their general level of satisfaction with the telehealth service of the Honorio Delgado Regional Hospital of Arequipa (N=377).

^a^ Mean (standard deviation); ^b^ Other provinces of the Arequipa region than the province of Arequipa; ^c^ Nurse, obstetrician, psychologist; ^d^ Mann-Whitney U;

^e^ Chi-square test; ^f^ Fisher's exact test

### Maturity level of the telehealth system

Overall, 32% of the items were found to be at the null level, 40.8% at the initiated level, 25.2% at the advanced level, and 2% at the ready level. Of the six evaluated categories, the regulatory aspects (Standards and procedures for the provision of telemedicine services) and organizational readiness (Bases that identify issues to be resolved before moving forward with telemedicine services) were mainly found at the advanced level, while the category of specialized knowledge (additional knowledge to benefit effective implementation) was found mostly at the null level (Figure 2). The complete assessment can be found in the supplementary material, Annex 5.

## DISCUSSION

The satisfaction level of the nonphysician HCP was higher than that of medical staff regarding clinical decision making via teleconsultation, with appointment outcome, with the "Teleatiendo" platform, and with the perceived satisfaction of their patients. In contrast, a systematic review reports that physicians have high levels of acceptability, feasibility, and compliance with the telehealth system and that their patients seemed very satisfied with telehealth care ^8^; however, these studies were characterized by the use of video teleconferencing. The low satisfaction of the physicians in our study may be due to the fact that they need physical examination to achieve an adequate evaluation, which is not possible when consultations are made by telephone calls. In this sense, the higher satisfaction of the non-physician HCP would be explained by the nature of their work, which is centered on tele-guidance and telemonitoring modalities, in which the telephone call is adequate or sufficient. This situation could improve if video calls were implemented in the telehealth service, since at least observation (physical examination) would be available as a basis for establishing a better clinical judgment.

Overall patient satisfaction was lower compared to studies in Latin America, which reported higher satisfaction (96% and 92.2%) ^15^^-^^17^, as well as to studies from other continents ^8^; this could be explained by being the only type of appointment available, differences in the health system and/or greater coverage of patients' health care expectations in other countries. The higher satisfaction level was found in patients attended by the pediatrics service, followed by gynecology and obstetrics. This could be due to the advantages of telehealth for these specialties, however, the desire for future telehealth appointments would be significantly affected by their digital experience, the perceived need for physical contact, time saved in travel and access to health providers ^18^. There was a higher level of dissatisfaction among patients attended by the surgery service, which could be due to the lack of video-call appointments ^19^.

The most used modality of "Teleatiendo" was telecounseling (83.0%), which is similar to what was reported by the Peruvian telehealth network ^20^. On the other hand, our results differ from what was reported in Spain, where the most used modality was telemonitoring (65.9%) ^21^, mainly for chronic patients, in whom telehealth seems to be more effective and compatible ^22^. We found that the "Teleatiendo" modality differs with the reported "consultation motive;" this may be due to the change of the appointment, from the time it is requested to the final service required by the patient and registered in the "Teleatiendo" system.

Satisfaction with the "follow-up" as a consultation motive (82.0%) was similar to that reported in Portugal and Spain, probably due to a higher frequency of appointments and a better relationship with the HCP ^21^^,^^23^. Likewise, it seems that older adult patients show less interest in telehealth follow-up ^21^, so it is necessary to create strategies to increase the satisfaction of this population and improve care according to their needs. The level of dissatisfaction of patients with the first appointment (22.3%) and with previous appointments (22.1%) was high, in contrast to the level of dissatisfaction of less than 7% during the first appointment reported by a previous study ^23^, this would be due to the unmet expectations of patients regarding the need for an examiner at the first appointment. Previous studies suggest models that allow a first face-to-face appointment and subsequent telehealth care ^22^, a proposal that should be examined in the future.

Telehealth - and digital health - can overcome geographical boundaries and contribute to improve the quality of care, accelerating progress towards universal health coverage ^24^. But this will only be achieved by bridging the gaps between digital services, healthcare providers and patients. Some of the digital gaps are: digital illiteracy, limited access to broadband (high-speed) internet, affordability of devices (smartphones, tablets, and computers), and difficult recruitment of patients from rural areas ^25^. Some of these gaps were evident in this study. The number of patients requiring support from a family member for telehealth appointments (more than 28%) is higher than in other studies (18% in Spain and 20% in Korea) ^9^^,^^26^. There was also limited use of technology and the Internet, since only 1.9% of patients made appointments via the Internet and 54.7% of patients made appointments by telephone, which takes a long time to materialize due to waiting times. This differs from other international studies, where appointments were mainly scheduled through the Internet, which showed a higher level of satisfaction ^27^.

Satisfaction with the telehealth service provides information on whether medical care is being provided correctly and whether it meets users' expectations. It is important for decision makers to consider patient and healthcare personnel satisfaction in the standardization of action plans for telehealth implementation, improvement, and follow-up ^28^.

There is currently no standard methodology for assessing the maturity status of a telehealth system. The Telehealth Readiness Assessment Tool (TRA) ^29^ assesses domains similar to those considered in the PAHO maturity level measurement tool, but the digital environment and regulatory operations are not included.

The items regarding regulatory aspects and organizational readiness were found to be in an advanced or ready status, indicating that the telehealth service implementation had started partially and safely ^14^. On the other hand, the items "specialized knowledge," "human resources," "digital environment" and "processes", were in a null or initiated status, which reflects the need to plan focused actions, explained in part by the hasty implementation of digital applications in user care due to COVID-19, and the lack of standardized regulations and guidelines in the field ^4^^,^^30^. International experts mention that there are factors associated with success that need to be considered, which are: the development of technical frameworks for action based on international standards, trained health workers and digitally literate health users ^4^, the latter two are scarce in the HRHD telehealth system.

The present study is the first to evaluate user satisfaction and the maturity level of a telehealth service implemented in a hospital in Peru, unlike other Latin American studies that studied only one service or specialty. 

The main limitation for this study was the fact that the Glasser *et al*. instrument has not been validated; however, it was chosen because it has been used in previous studies to establish similar comparisons, and it was one of the instruments that showed the best performance compared to other instruments used in previous research, which had questions aimed at patients and not health personnel. The instruments used were designed for consultations by video call, but in the HRHD the consultations were by telephone, so some questions were adapted without substantially changing the purpose of the questions. In addition, there was a high non-response rate during sampling. Only one hospital was included, so the external reproducibility of this study is low; however, it provides a realistic approximation of what would be happening in other centers of the Peruvian National Telehealth Network.

In conclusion, the degree of satisfaction of non-medical health personnel was moderately high, in contrast to the low satisfaction of medical personnel. Patients showed a moderately high degree of satisfaction with telehealth, although barriers such as digital illiteracy and technological limitations are still obstacles to telehealth in Peru. The maturity level of telehealth implementation in HRHD is more oriented towards the initiated level, so incremental improvements will be necessary. Future research should study telehealth services in other contexts at the national level. In this regard, we recommend studies with designs that delve deeper into the research of satisfaction such as those on associated factors as well as qualitative studies. Further post-pandemic studies will be necessary to assess the evolution of patient and HCP satisfaction, as well as the status of the implementation or maturity of the telehealth system.
