# Supplementary material for: Satisfaction of healthcare workers and patients regarding telehealth service in a hospital in Peru
Source: Rev Peru Med Exp Salud Publica. 2022 Dec 30;39(4):415–24. doi: 10.17843/rpmesp.2022.394.11287 (PMC11397586; doi:10.17843/rpmesp.2022.394.11287)
Supplement: Supplementary material. — Available in the electronic version of the RPMESP. [file rpmesp-39-04-11287-s001.docx]

**MATERIAL SUPLEMENTARIO**

**Anexo 1.** Características de las atenciones del servicio de telesalud del HRHD desde mayo del 2021 a enero 2022. Ante la emergencia sanitaria, el HRHD tuvo que implementar el sistema de telesalud de forma acelerada para dar respuesta a la pandemia lo más pronto posible. Se empezó a operar en agosto del 2020. El primer registro digital que se tiene en la plataforma “Teleatiendo” corresponde al mes de mayo del 2021 e inició con 4951 atenciones. La mayor cantidad de atenciones fue en enero del 2022 (10714 pacientes). En el **anexo 1a**, se muestra la evolución de las atenciones, mostrando que se redujeron ligeramente entre los meses de septiembre a diciembre del 2021. En el **anexo 1b**, la mayor cantidad de pacientes que fueron atendidos tenían seguro de salud SIS, y en el **anexo 1c**, hubo una notable disminución de atenciones mensuales a pacientes de Arequipa-Perú desde el mes de mayo del 2021 (77,9%) para incrementar las atenciones en otras provincias para enero del 2022 (47,1%).

**ANEXO 2:** Sugerencias y comentarios del personal de salud médico que respondieron cualitativamente al finalizar la encuesta (N=35)

| **Conclusión** | **Comentario representativo** | **Cantidad de comentarios** |
| --- | --- | --- |
| La telesalud no ayuda en el campo quirúrgico | “En el campo quirúrgico necesitamos ver y examinar al paciente y no podemos hacer nada, apenas preguntar cómo se siente, que le duele. Cuando pedimos que nos manden una foto muchas veces no se ve bien” | 8 |
| Cada actualización de la plataforma Teleatiendo genera confusión en los médicos. | “En cada actualización de la plataforma Teleatiendo cambian algunas cosas que hacen que confunden” | 6 |
| La plataforma Teleatiendo no se encuentra sincronizada con las historias físicas de los pacientes. | “Todo lo que hemos atendido no está en la historia hospitalaria, es como si no hubiéramos dado tratamiento al paciente” | 5 |
| Hay problemas al generar recetas y con los códigos CIE 10. La plataforma brinda opciones limitadas. | “Deberían mejorar la plataforma, por ejemplo, en la primera versión de la plataforma, las recetas yo las podía escribir y ahora no se puede. No puedo hacer recetas específicas para el paciente” | 5 |
| La plataforma Teleatiendo colapsa en los horarios de la mañana debido a la cantidad de atenciones que se generan. | “La plataforma Teleatiendo es lenta cuando yo atiendo a pacientes debo hacerlo a través de otros sistemas y después paso los datos es decir para siempre saturada la plataforma de Teleatiendo” | 3 |
| Debería de darse al menos una consulta presencial a los pacientes antes de ser atendidos remotamente. | “Pienso que la mayoría de pacientes que son vistos por teleconsulta deberían tener al menos una consulta presencial. La mayoría de veces hacemos seguimiento y son pacientes que nunca hemos visto de manera presencial” | 3 |
| Debería hacerse uso de videollamada y existir un medio para que los pacientes envíen sus fotos | “La teleconsulta mejoraría si se hiciera usa de videollamada no solo de llamada” | 2 |
| No hay un buen registro de datos de los pacientes por ejemplo en el número de DNI o número de celular” | “Hay un mal acopio de información. La información de los pacientes es mala El número, nombre es equivocado. Cuando se atiende venezolanos nunca acepta el carnet de extranjería” | 1 |
| Están saturando el horario de los médicos sus horarios de trabajo | “Nos están saturando de pacientes, nos programan pacientes en días libres” | 1 |

**ANEXO 3:** Sugerencias y comentarios del personal de salud no médico que respondieron cualitativamente al finalizar la encuesta (N=27)

| **Conclusión** | **Comentario representativo** | **Cantidad de comentarios** |
| --- | --- | --- |
| Falta de coordinación entre centros de salud y DIRESA. | “Recomienda que la información debe ser actualizada, si todas las páginas de bases de datos de los centros de salud actualizaran los datos, se manejaría mejor al paciente” | 9 |
| Los pacientes y sus familiares desconfían cuando reciben una llamada del hospital por falta de información acerca de la telemedicina. | “La comunidad no estaba informada sobre este tipo de trabajo remoto y la gente no quería colaborar” | 7 |
| Hay una falta de capacitaciones para todo el personal de salud acerca del uso de tecnología y telemedicina. | “Todo el personal de Salud debería saber sobre telemedicina, antes solo eran los médicos que la utilizaban, pero ahora otros servicios debieron aprender aceleradamente sobre el uso de la telemedicina” | 5 |
| Deficiencias con la plataforma de “Teleatiendo”. | “Le hubiera gustado que el servicio de teleorientación cuente con material multimedia para hacer más práctico las indicaciones y lograr una mejor comprensión” | 4 |
| Mal uso de la línea 113 | “La línea 113 está siendo mal utilizada, las personas llaman para preguntar "En donde, cuando me vacunado" cuando esa información ya fue dada por la página del ministerio, pacientes piensan que este es un multiservicio en el que solicitan incluso el envío de médicos a domicilio (principalmente personas de estrato social alto) según refiere” | 1 |
| Lado positivo del telemonitoreo. | “El trabajo remoto sirve mucho a los familiares del paciente, respecto a la forma de comprender la enfermedad, medicación (especialmente en pacientes psiquiátricos)” | 1 |

**ANEXO 4:** Sugerencias y comentarios de los pacientes que respondieron cualitativamente al finalizar la encuesta (N=35)

| **Conclusión** | **Comentario representativo** | **Cantidad de comentarios** |
| --- | --- | --- |
| Preferencia de atención presencial, aunque se reconoce la efectividad de la telemedicina en la pandemia | “Prefiero que sea presencial la atención” | 5 |
| Los principales factores identificados son: el número de veces que se tiene que llamar para ser atendido (hasta 15 veces), atención poco cordial. | “La única recomendación es mejorar el sistema de cómo sacar citas, tiene que llamar muchas veces para obtener una cita” | 14 |
| Falta de efectividad y rapidez en la atención de especialistas | “Sería buena si las respuestas fueran inmediatas; muchas veces los médicos no informan sobre su enfermedad, le dejan más dudas” | 5 |
| Los pacientes identifican como principales dificultades: falta de instrucciones en el uso del sistema, falta de actualización rápida y continua de la base de datos que incluye: programación de operaciones, recetas. | “Deberían de dar más información acerca de las llamadas” | 5 |
| Mala identificación y trato del personal de salud | “Deberían ser más empáticos los doctores” | 4 |
| Se sugiere la capacitación del adulto mayor para con el uso del sistema, así como otra modalidad de contacto, ya que la llamada telefónica dificulta la atención. | “Para los adultos mayores debería haber algún taller, u otra modalidad” | 2 |
|  | “Mal trato por los médicos, salía con los mismos síntomas” | 7 |
| Falta orientación y paciencia hacia los pacientes |  |  |
| Solo un comentario, bue positivo, respecto a la atención en general. | “Forma muy grata” | 1 |
| Dificultad de los pacientes en la comunicación de los síntomas hacia el médico. | Resulta difícil de expresar tus síntomas al médico | 2 |
| Falta de información y atención brindada. | “No le dicen nada, no le dan tratamiento; los Dres. le dicen que le llamaran y no le llaman, sacan cita de nuevo, pero no les atienden” | 1 |

**Anexo 5:** Herramienta de medición del nivel de madurez de las instituciones de salud para implementar servicios de telemedicina

| **Ítem** | **Pregunta** | **Nulo** | **Iniciado** | **Avanzado** | **Listo** |
| --- | --- | --- | --- | --- | --- |
|  |  | **1** | **2** | **3** | **4** |
| **I. PREPARACIÓN ORGANIZACIONAL** | | | | | |
| 1 | ¿Está la alta gerencia decidida a ofrecer servicios de telemedicina? |  |  | x |  |
| 2 | ¿Se comprende claramente qué servicios pueden ofrecerse a través de la telemedicina? |  | x |  |  |
| 3 | ¿Se han identificado los servicios que se ofrecerán vía telemedicina? |  |  | x |  |
| 4 | ¿Se dispone de presupuesto para ofrecer servicios de telemedicina? |  |  | x |  |
| 5 | ¿Está capacitado el personal informático para prestar servicios de apoyo a la telemedicina? |  |  | x |  |
| 6 | ¿Permiten los marcos regulatorios nacionales o locales la implementación de servicios de telemedicina? |  |  |  | x |
| 7 | ¿Cuenta la institución con acceso estable a internet? |  | x |  |  |
| 8 | ¿Cuenta la institución con algún programa de telemedicina en funcionamiento? |  |  | x |  |
| 9 | ¿Cuenta la institución con alguna iniciativa de uso de mensajería instantánea o de texto para la promoción de la salud? | x |  |  |  |
| 10 | ¿Tiene la institución alguna experiencia en la prestación de servicios a través de consultas virtuales? |  |  | x |  |
| 11 | ¿Tiene la institución alguna experiencia en el seguimiento remoto de pacientes? |  |  | x |  |
| 12 | ¿Podría extenderse el financiamiento de los servicios de telemedicina más allá de la planificación y el período inicial y el piloto, para convertirse en un modelo sostenible? |  | x |  |  |
| 13 | ¿Está capacitado el personal médico para prestar servicios de telemedicina? Véase el apartado «Recursos humanos» más adelante para obtener más detalles |  | x |  |  |
| 14 | Si la respuesta anterior es 1 o 2, ¿Se han establecido opciones de formación y capacitación en telemedicina? |  | x |  |  |
| 15 | ¿Cuenta la institución con la infraestructura necesaria para prestar servicios de telemedicina? |  |  |  |  |
|  | 15.a. Espacio adecuado | x |  |  |  |
|  | 15.b. Suministro estable de electricidad |  | x |  |  |
|  | 15.c. Iluminación aceptable. |  |  | x |  |
|  | 15.d. Equipos de apoyo. | x |  |  |  |
| 16 | ¿Se ha designado a una persona para actuar como responsable de los servicios de telemedicina? |  |  | x |  |
| 17 | ¿Está el personal médico de acuerdo con ofrecer servicios de telemedicina? |  |  | x |  |
| 18 | ¿Se ha trabajado en la resistencia al cambio de rutinas en las que los médicos se sienten seguros y confortables por una nueva y desconocida que implica cierto grado de incertidumbre inicial? | x |  |  |  |
| 19 | Si su respuesta anterior es 1 o 2, ¿Se puede solucionar este problema con diálogos participativos? |  |  | x |  |
| 20 | ¿Se ha establecido algún mecanismo de incentivación para usar la telemedicina? | x |  |  |  |
| 21 | ¿Conoce el personal médico las prácticas de privacidad y seguridad basadas en los principios éticos y legales vigentes? |  | x |  |  |
| 22 | ¿Se ha definido la carga de trabajo que supone poner en marcha este tipo de programas en el entorno actual? |  | x |  |  |
| 23 | ¿Se cuenta con el apoyo de alguna institución especializada en servicios de telemedicina? |  |  | x |  |
| 24 | ¿Se ha comunicado al personal de la institución la intención de implementar o fortalecer los servicios de telemedicina? |  |  | x |  |
| 25 | ¿Se ha comunicado a los potenciales beneficiarios de los servicios de telemedicina su apertura o fortalecimiento? |  |  | x |  |
| 26 | ¿Se han cambiado las agendas de la atención a los pacientes por la necesidad de consultas no presenciales? |  |  | x |  |
| 27 | ¿Qué nivel de aceptación de los servicios de telemedicina se espera de sus potenciales beneficiarios? |  |  | x |  |
| 28 | ¿Existe alguna barrera cultural o lingüística que pueda ocasionar dificultades durante la prestación de los servicios de telemedicina? |  | x |  |  |
| 29 | ¿Se conoce el nivel de conectividad de los potenciales pacientes? |  |  | x |  |
| 30 | ¿Se conoce el nivel de alfabetización digital de los potenciales pacientes? |  | x |  |  |
| 31 | ¿Se han establecido mecanismos de gobernanza? |  |  |  | x |
| 32 | ¿Se han establecido mecanismos de evaluación continua? | x |  |  |  |
| **2. PROCESOS** | | | | | |
| 33 | ¿Se han definido las funciones, los roles y las responsabilidades asociadas a los servicios de telemedicina para todo el personal que estará involucrado? |  |  | x |  |
| 34 | ¿Se han definido las funciones, los roles y las responsabilidades relacionadas con los servicios de telemedicina para todo el personal administrativo? |  | x |  |  |
| 35 | ¿Existen procesos definidos para abordar las consideraciones de seguridad del paciente y de responsabilidad legal institucional? |  | x |  |  |
| 36 | ¿Existen procesos definidos para garantizar la seguridad, la confidencialidad, y la copia de los datos y de la información generadas durante los servicios de telemedicina? |  | x |  |  |
| 37 | ¿Existen procedimientos para registrar el estado de satisfacción del paciente de los servicios de telemedicina? |  | x |  |  |
| 38 | ¿Existen procedimientos para notificar incidentes o sucesos adversos ocurridos durante las consultas de telemedicina? | x |  |  |  |
| 39 | ¿Existen procedimientos estandarizados para comunicar y documentar posibles fallas técnicas durante una consulta que pudieran afectar a los resultados clínicos? | x |  |  |  |
| 40 | ¿Existen procedimientos formales para obtener el consentimiento informado de los pacientes de manera remota? | x |  |  |  |
| 41 | ¿Existen procedimientos o herramientas para que el personal médico y los pacientes puedan compartir sus preocupaciones, sugerencias o comentarios sobre cómo se va desarrollando el programa de telemedicina? | x |  |  |  |
| 42 | ¿Existe una estrategia y un plan operativo que guíen a los proveedores de servicios de salud para que opten por las teleconsultas ambulatorias y al monitoreo remoto de pacientes? |  | x |  |  |
| 43 | ¿Existen mecanismos de comunicación para informar y educar a la población sobre el uso recomendado de la telemedicina? | x |  |  |  |
| 44 | ¿Existe un procedimiento o plan de emergencia para cuando los médicos que practican la telemedicina consideren que se debe derivar al paciente a un centro de cuidados intensivos? |  | x |  |  |
| **3. ENTORNO DIGITAL** | | | | | |
| CONEXIÓN A INTERNET Y CONECTIVIDAD | | | | | |
| 45 | ¿Se dispone de un servicio de conexión a internet fijo y estable? |  | x |  |  |
| 46 | ¿Permite el ancho de banda ofrecer servicios de telemedicina sin que otros servicios se vean afectados? |  | x |  |  |
| 47 | ¿Se sabe cómo calcular el ancho de banda necesario para prestar servicios de telemedicina? | x |  |  |  |
| 48 | ¿Se dispone de los equipos mínimos necesarios (hardware)? |  | x |  |  |
| 49 | Si la respuesta anterior es 1 o 2 ¿Se dispone del presupuesto que hace falta para adquirir los equipos necesarios? | x |  |  |  |
| 50 | ¿Se dispone de apoyo técnico en la propia institución para resolver problemas relacionados con la conectividad? | x |  |  |  |
| 51 | ¿Existe un plan de ciberseguridad? | x |  |  |  |
| 52 | ¿Existe un acceso a guías técnicas de apoyo para temas de conectividad? | x |  |  |  |
| 53 | ¿Existe un plan de contingencia para fallas de equipos o de conectividad? | x |  |  |  |
| 54 | ¿Se ha considerado qué impacto pueden tener los nuevos servicios de telemedicina sobre la infraestructura tecnológica actual? |  | x |  |  |
| APLICACIONES (SOFTWARE PARA GESTIÓN DE REGISTROS MÉDICOS, PORTALES DE PACIENTES, ETC.) | | | | | |
| 55 | ¿Existe un sistema de registro electrónico de pacientes? |  |  | x |  |
| 56 | ¿Existe con un portal de pacientes? |  |  | x |  |
| 57 | ¿Se sabe qué software o soluciones informáticas son necesarios para ofrecer servicios de telemedicina? |  | x |  |  |
| 58 | ¿Existe procedimientos estándares de operación para la gestión de datos y procesos relacionados con telemedicina? |  | x |  |  |
| 59 | ¿Existen guías sobre: |  |  |  |  |
|  | 59.a. seguridad del paciente? | x |  |  |  |
|  | 59.b. privacidad y confidencialidad de los datos? |  | x |  |  |
| 60 | ¿Cuál es el nivel de interoperabilidad entre los diferentes sistemas y bases de datos de los servicios de telemedicina? |  | x |  |  |
| 61 | ¿Cuenta con términos de referencia para la adquisición de soluciones informáticas? |  | x |  |  |
| 62 | ¿Se ha considerado si la solución informática para telemedicina se integrará con otros sistemas y procesos existentes, como los de registros médicos, portales de pacientes, mensajería, etcétera? |  | x |  |  |
| 63 | ¿Existen procedimientos estándares de operación para la gestión de datos y procesos relacionados con la atención de pacientes? |  | x |  |  |
| 64 | ¿Las plataformas para registros médicos usados en servicios de telemedicina tienen capacidad de incluir copias de todas las comunicaciones electrónicas relacionadas con el paciente? | x |  |  |  |
| APLICACIONES (SOFTWARE ADMINISTRATIVO PARA FACTURACIÓN, PAGOS, CONTROL HORARIO, ETC.) | | | | | |
| 65 | ¿Están preparadas las plataformas de gestión administrativa para acompañar la implementación de servicios de telemedicina? |  | x |  |  |
| EQUIPO TÉCNICO (HARDWARE Y OTROS DISPOSITIVOS) | | | | | |
| 66 | ¿Existe un inventario de todo el equipamiento técnico, que incluya la marca, el modelo, el tiempo de funcionamiento y el número de serie? | x |  |  |  |
| 67 | ¿Existe una ubicación segura para almacenar el equipo cuando no se está utilizando? | x |  |  |  |
| 68 | ¿Existe un programa de mantenimiento del equipamiento técnico? | x |  |  |  |
| 69 | ¿Se ha considerado qué capacidad tecnológica de almacenamiento y seguridad son necesarias para documentar y registrar los encuentros presenciales? |  | x |  |  |
| 70 | ¿Se dispone del apoyo técnico de especialistas en tecnologías de la información? |  | x |  |  |
| 71 | ¿Existe un programa de renovación del equipamiento técnico? | x |  |  |  |
| **IV. RECURSOS HUMANOS** | | | | | |
| PERSONAL DE SALUD | | | | | |
| 72 | ¿Hay personal disponible para participar en el programa de telemedicina? |  |  | x |  |
| 73 | ¿Se ha determinado con claridad qué personal trabajará en los servicios o especialidades de telemedicina? |  |  | x |  |
| 74 | ¿Se han determinado las capacidades mínimas de las personas que trabajarán en los servicios de telemedicina en los distintos servicios de telemedicina? |  | x |  |  |
| 75 | ¿Se considera desarrollar un plan de capacitación acelerado para el personal menos experto en tecnologías de información? | x |  |  |  |
| 76 | ¿Existe acceso a programas de capacitación rápida de otras instituciones, en caso necesario? |  | x |  |  |
| 77 | ¿Está previsto prestar servicios de telemedicina fuera del horario de atención presencial? | x |  |  |  |
| 78 | ¿Cuál es el nivel de capacitación del personal para prestar servicios de telemedicina? |  | x |  |  |
| PERSONAL DE TECNOLOGÍAS DE LA INFORMACIÓN | | | | | |
| 79 | ¿Se cuenta con personal de tecnologías de la información propio que apoye a los servicios de telemedicina? |  | x |  |  |
| 80 | Si la respuesta anterior es inferior que 3, ¿está claro cuál debe ser el perfil de las personas que atenderán los servicios de telemedicina? |  | x |  |  |
| 81 | ¿Existe personal capacitado para realizar un análisis crítico de cualquier equipo y de las tecnologías de la información que se deban adquirir? |  | x |  |  |
| 82 | ¿Existe personal con la experiencia necesaria para negociar con proveedores la compra o contratación de tecnologías de información o de servicios de apoyo? | x |  |  |  |
| 83 | ¿Cuál es el nivel de capacitación del personal informático de la institución para prestar servicios de telemedicina? |  | x |  |  |
| **V. ASPECTOS NORMATIVOS** | | | | | |
| 84 | ¿Están completamente claros todos los aspectos jurídicos asociados a la prestación de servicios de telemedicina? |  |  | x |  |
| 85 | ¿Existe un proceso para mantener actualizado al personal que presta servicios de telemedicina sobre posibles cambios en las reglamentaciones, estatutos, políticas federales y estatales, y la legislación relacionada con los servicios de telemedicina? | x |  |  |  |
| 86 | ¿Cuenta la institución con una asesoría legal interna? ¿Tiene acceso a un servicio de consultoría jurídica especializada para pedir consejo a expertos en cuestiones de legalidad, ética, privacidad y seguridad? |  |  | x |  |
| 87 | ¿Se sabe a ciencia cierta que los pacientes se encuentran en los mismos límites geográficos (estado, provincia o municipio) que la institución que presta los servicios de telemedicina? |  |  | x |  |
| 88 | ¿Se conocen bien los aspectos de mala praxis relacionados con los servicios de telemedicina? | x |  |  |  |
| 89 | ¿Se ha establecido un procedimiento para obtener y documentar el consentimiento del paciente antes de que participe en una visita de telemedicina? | x |  |  |  |
| 90 | ¿Existe un marco regulatorio para autorizar, integrar y reembolsar la telemedicina en la prestación de atención a todos los pacientes, en particular en situaciones de emergencia y brotes? |  |  | x |  |
| 91 | ¿Se necesitan nuevas reglamentaciones o marcos técnicos jurídicos para implementar servicios de telemedicina? |  | x |  |  |
| **VI. CONOCIMIENTO ESPECIALIZADO** | | | | | |
| 92 | Indicadores para proyectos de telemedicina como herramienta para reducir las inequidades en salud. | x |  |  |  |
| 93 | Protocolos de atención por vía telemedicina. |  | x |  |  |
| 94 | Estándares de informática en salud e interoperabilidad. | x |  |  |  |
| 95 | Gestión del cambio. | x |  |  |  |
| 96 | Arquitectura de información en salud pública. |  | x |  |  |
| 97 | Preparación tecnológica en salud pública | x |  |  |  |
| 98 | Gobernanza de datos en salud pública. |  | x |  |  |
| 99 | Marco de implementación de la telemedicina |  | x |  |  |
|  | **TOTAL** | 33 | 42 | 26 | 2 |
