# Supplementary material for: Satisfaction of healthcare workers and patients regarding telehealth service in a hospital in Peru
Source: Rev Peru Med Exp Salud Publica. 2022 Dec 30;39(4):415–24. doi: 10.17843/rpmesp.2022.394.11287 (PMC11397586; doi:10.17843/rpmesp.2022.394.11287)
Supplement: Translation

# Translation: Satisfacción del personal de salud y pacientes sobre un servicio de telesalud de un hospital de Perú

**Authors:** Fernanda Barriga-Chambi, Fabricio Ccami-Bernal, Alberto Luciano Alarcon-Casazuela, Julissa Copa-Uscamayta, Jeremy Yauri-Mamani, Brenda Oporto-Arenas, Cender Udai Quispe-Juli

**Affiliations:** 1 Universidad Nacional de San Agustín de Arequipa, Arequipa, Perú.; 2 Sociedad Científica de Estudiantes de Medicina Agustinos (SOCIEMA), Arequipa, Perú.; 3 Universidad Peruana Cayetano Heredia, Lima, Perú.

**Keywords:** Telemedicina, Consulta remota, COVID-19, Satisfacción del Paciente, Personal de Salud

## Abstract

**Objetivos.:**

Evaluar el nivel de satisfacción con el servicio de telesalud del Hospital III Regional Honorio Delgado (HRHD) por parte del personal de salud y los pacientes atendidos, así como el nivel de madurez de implementación de dicho servicio.

**Materiales y métodos.:**

Estudio observacional transversal llevado a cabo desde octubre a diciembre del 2021. La satisfacción del personal de salud y de los pacientes fueron evaluadas con la encuesta de Glaser *et al*. y el cuestionario Telemedicine Satisfaction Questionnaire (TSQ), respectivamente. El nivel de madurez del servicio se evaluó mediante la herramienta de medición del nivel de madurez de las instituciones de salud para implementar servicios de telemedicina de la Organización Panamericana de la Salud.

**Resultados.:**

Se obtuvieron 129 respuestas del personal de salud. El número de profesionales no médicos satisfechos con el servicio de telesalud fue mayor al de médicos (72,5% vs 18,3%). De los 377 pacientes, el 77,6% manifestó estar satisfecho con el servicio. Con respecto al nivel de madurez, el servicio de telemedicina del HRHD cuenta con 32% de ítems en estado nulo, 40,8% en iniciado, 25,2% en avanzado y 2% en listo.

**Conclusiones.:**

La satisfacción de los médicos fue menor a la de los otros profesionales de la salud. Los pacientes reportaron una satisfacción moderadamente alta. La implementación del servicio de telesalud en el HRHD se orienta hacia un nivel nulo o iniciado. Es importante que los tomadores de decisiones consideren la satisfacción de usuarios durante la implementación y seguimiento de los servicios de telesalud.

## INTRODUCCIÓN

La pandemia por COVID-19 ha desafiado la capacidad de atención presencial de los sistemas de salud, obligándolos a rediseñar y/o implementar otras modalidades para la atención del paciente mediante la telesalud ^1^. Según la OMS, la telesalud es la prestación de servicios en salud a distancia que utiliza Tecnologías de la Información y la Comunicación (TIC) para el diagnóstico, tratamiento y prevención de enfermedades ^2^. Presenta ventajas como mayor acceso, optimización de recursos, minimización de costos, menor riesgo de contagio, entre otras ^3^. Por otro lado, surgen preocupaciones sobre vacíos normativos, infraestructura de TIC, capacitación del personal, brecha digital, seguridad y privacidad, entre otras que constituyen barreras para su implementación ^4^. La implementación y sostenibilidad de estos servicios dependen en gran parte de la percepción y satisfacción de pacientes y personal de salud (PS), ya que son la principal fuente de información para conocer si la atención médica se brinda de manera correcta y si cumple con sus expectativas ^5^.

La satisfacción del paciente se ve influenciada por varios factores, como la prestación del servicio (disponibilidad de una especialidad, frecuencia de atención, obtención de la información, tratamiento ambulatorio o de hospitalización), la relación médico-paciente y factores tecnológicos (acceso al sistema, ausencia de problemas técnicos) ^1^. Estudios previos hallaron una alta satisfacción en pacientes, que variaba entre 68% a 100% ^6^^,^^7^. Pocos estudios han evaluado la satisfacción del personal de salud, (satisfacción reportada entre 64%-81%) ^8^. Se ha encontrado, que la satisfacción de los pacientes con el servicio de telesalud a través de llamadas telefónicas fue mayor que la del personal de salud ^9^. A pesar del desarrollo de iniciativas de telesalud durante la pandemia de COVID-19, pocos estudios en América Latina y el Caribe (ALC) han evaluado la satisfacción de pacientes y/o del personal de salud ^10^, no hay estudios publicados en revistas peruanas indizadas.

Pocos países en ALC cuentan con políticas y reglamentos en telesalud bien establecidos, por lo que conocer la satisfacción de pacientes y del personal de salud - como un elemento de retroalimentación - permitiría redirigir la regulación para mejorar la atención en salud a través de la telesalud ^11^. El objetivo del presente estudio fue evaluar el grado de satisfacción del personal de salud y los pacientes atendidos por el servicio de telesalud en el Hospital Regional III Honorio Delgado (HRHD) de Arequipa, Perú. El objetivo secundario fue evaluar el nivel de madurez de implementación de telesalud en el HRHD.

**MENSAJES CLAVE**

Motivación para realizar el estudio: es importante conocer la satisfacción de los usuarios con respecto a la rápida implementación y creciente uso de la telesalud debido a la pandemia de la COVID-19. Existen pocos estudios en Latinoamérica y ninguno en Perú.

Principales hallazgos: la satisfacción del personal de salud no médico es moderadamente alta, contrastada con la baja satisfacción del personal médico. La satisfacción de los pacientes es moderadamente alta. La implementación de la telesalud en el Hospital III Regional Honorio Delgado se encuentra en un nivel nulo o iniciado, y reflejaría la situación en varios centros de la Red Nacional de Telesalud.

Implicancias: conocer el nivel de satisfacción de los usuarios permitirá la estandarización de planes de acción, así como, mejorar la implementación, perfeccionamiento y seguimiento de los servicios de telesalud para brindar una mejor calidad de atención sanitaria.

## MATERIALES Y MÉTODOS

### Diseño y lugar del estudio

El diseño del presente estudio fue observacional transversal, con un enfoque descriptivo. El estudio se llevó a cabo en el HRHD de Arequipa, perteneciente a la Red Nacional de Telesalud, cuyo servicio fue implementado de forma acelerada en agosto del 2020 debido a la pandemia de la COVID-19. El HRHD utiliza la plataforma "Teleatiendo" para gestionar las citas, registrar datos de la atención y otros procesos relacionados. El HRHD ha sido reconocido en dos oportunidades como una las de instituciones prestadoras de servicios de salud con mayor cantidad de atenciones en "Teleatiendo" a nivel nacional. Es importante precisar que la totalidad de las atenciones se realizan mediante llamadas telefónicas. El primer registro encontrado de la plataforma corresponde a mayo del 2021 con 4951 atenciones alcanzando un máximo de 10 714 en enero del 2022 (material suplementario, anexo 1). 

### Población y muestra

La población de estudio estuvo conformada por el personal de salud (PS) del HRHD que realizó atención mediante telesalud durante el periodo 2020-2021 y los pacientes atendidos por el servicio de telesalud del HRHD desde el 15 de octubre al 15 de diciembre del 2021. Se buscó la participación de la totalidad del PS. Se utilizó un muestreo aleatorio estratificado de pacientes. Para calcular el tamaño de muestra se tomó como referencia el total de pacientes atendidos registrados en la plataforma "Teleatiendo" en los meses de agosto y septiembre (20 828 pacientes). El tamaño de muestra calculado fue de 377 pacientes, con un nivel de confianza del 95% y una frecuencia porcentual de evento de interés del 50%. Los estratos considerados fueron: teleorientación, telemonitoreo y teleconsulta (modalidades de atenciones en "Teleatiendo") con una razón de 28/5/1. La selección de pacientes fue aleatoria, semanal y proporcional a los estratos durante el tiempo de estudio y se hizo hasta alcanzar el tamaño mínimo muestral calculado. 

Se incluyó al PS del HRHD que realizó telesalud durante el periodo 2020-2021 y los pacientes atendidos por el servicio de telesalud del HRHD del departamento de Arequipa que tuvieron al menos una cita registrada en la plataforma "Teleatiendo" del HRHD. Se excluyó al PS con menos de seis meses de experiencia de atención por telesalud, se excluyeron a los pacientes que negaron haber sido atendidos por el servicio de telesalud del HRHD.

### Variables e instrumentos

La satisfacción del PS fue evaluada con la encuesta de Glasser *et al*. ^12^, operacionalizada mediante una escala de Likert. Las alternativas de las dos primeras preguntas fueron: muy de acuerdo, de acuerdo, neutro, en desacuerdo y muy en desacuerdo; para las tres últimas preguntas fue completamente satisfecho, satisfecho, neutro, insatisfecho, completamente insatisfecho. Se consideró otras variables como: sexo, edad, profesión, tiempo ejerciendo la profesión (años), tiempo de experiencia en telesalud (meses), experiencia en telesalud previa a la pandemia, frecuencia de atención por telesalud (diario, semanal, mensual), percepción de la competencia del uso de la tecnología para telesalud (principiante, intermedio, avanzado).

La satisfacción de los pacientes fue evaluada con el cuestionario validado y ampliamente utilizado, *Telemedicine Satisfaction Questionnaire* (TSQ) ^13^, y fue operacionalizada mediante una escala de Likert en muy de acuerdo, de acuerdo, neutro, en desacuerdo y muy en desacuerdo; y la ultima pregunta sobre la satisfacción general se agrupó en dos categorías satisfecho (muy de acuerdo y de acuerdo) y no satisfecho (neutral, en desacuerdo y muy en desacuerdo). También se registraron variables sociodemográficas, seguro de salud, lugar de procedencia (provincia de Arequipa, otras provincias), acceso a Internet, modalidad en "Teleatiendo" (teleorientación, telemonitoreo y teleconsulta), personal que lo atendió (médico o no médico, el cual incluyó enfermero/a, obstetra, psicólogo/a), especialidad de unidad productora de servicios (medicina, cirugía, pediatría, ginecología y obstetricia e inmunizaciones) y motivo de la cita. Para la variable "motivo de cita", se revisaron las citas en "Teleatiendo" de cada paciente, y se clasificó según la actividad principal que se realizaba durante la cita (consejería, seguimiento o consulta), considerando las citas previas e indicaciones. Adicionalmente, al final de la encuesta al PS y a los pacientes se les hizo una pregunta libre sobre sus comentarios y sugerencias sobre la plataforma y la atención.

Para evaluar el nivel de madurez del sistema de teleconsulta del HRHD, se usó la herramienta de medición del nivel de madurez de instituciones de salud de la Organización Panamericana de salud (OPS), operacionalizada según el nivel de avance de la implementación de los servicios de telemedicina como: nulo (sin ninguna iniciativa), iniciado (con avances, pero lejos de lo necesario), avanzado (buenos avances) y listo (funciona en plena capacidad). Este instrumento tiene seis dimensiones: 1) preparación organizacional (bases que identifican temas a resolver antes de avanzar con los servicios de telemedicina), 2) procesos (conjunto de operaciones y funciones a considerar), 3) entorno digital (infraestructura tecnológica necesaria), 4) recursos humanos (capacidad institucional en las áreas de servicios de salud y TIC), 5) aspectos normativos (normas y procedimientos para la provisión de servicios de Telemedicina), 6) conocimiento especializado (conocimiento adicional para que beneficia una implementación efectiva) ^14^.

### Procedimientos

Se solicitó permiso al HRHD para hacer el estudio y acceder a la base de datos del PS y pacientes usuarios de telesalud durante octubre a diciembre del 2021. Las encuestas fueron realizadas a través de llamadas telefónicas por los investigadores y nueve colaboradores previamente entrenados. Para facilitar la aplicación de los instrumentos se estandarizó el lenguaje de las preguntas basado en una prueba piloto, luego de la cual se adaptaron los términos para una mejor comprensión. 

Se encuestó al PS, previa notificación sobre el estudio por parte del servicio de telesalud, fuera de su horario de trabajo, en caso de no respuesta se dejó un mensaje y se volvió a llamar en otro horario hasta en cinco oportunidades. Se encuestó a los pacientes mayormente durante la mañana y la noche, en caso de no respuesta, se llamó hasta dos veces. A cada encuestado se le explicó la finalidad del estudio para obtener su consentimiento verbal. Para reducir el sesgo de memoria, los pacientes fueron encuestados hasta dos semanas después de su atención. Los datos epidemiológicos y aquellos relacionados a la cita de telesalud, fueron completados con los datos de la plataforma "Teleatiendo", que se obtuvieron mediante una ficha de recolección.

La recolección de información relacionada al nivel de madurez, se hizo mediante tres visitas de campo a cargo de dos observadores (FBC, ALAC), quienes recabaron información mediante observación, diálogo con el personal de salud y administrativos, y la verificación de elementos probatorios en el servicio de telesalud, según el instrumento de la OPS. Las preguntas que no fueron absueltas mediante las técnicas establecidas previamente fueron resueltas mediante una entrevista con la secretaría y la jefa de la oficina de telesalud del HRHD. Los investigadores se reunieron y llegaron a un consenso sobre la puntuación final de cada indicador.

### Análisis estadístico

El almacenamiento y procesamiento de datos se hizo en una hoja de cálculos de Google (r), así como en Microsoft Excel 2019 (r). Se eliminaron las fichas con datos incompletos. Se calcularon las medidas de tendencia central y de dispersión (variables numéricas), y las frecuencias absolutas y relativas (variables categóricas). Se utilizó la prueba de chi cuadrado, prueba exacta de Fisher y U de Mann-Whitney según correspondió para comparar los grupos; se consideró un valor de p<0,05 como estadísticamente significativo. El análisis estadístico fue realizado con el programa estadístico STATA 16.0.

### Aspectos éticos

Se informó sobre el propósito del estudio a los participantes, luego se solicitó su consentimiento verbal para ser encuestados y grabados, no se brindó ningún incentivo. Los datos fueron anonimizados. Este estudio fue guiado por los principios de conducta responsable en investigación e integridad científica, y fue aprobado por el Comité Institucional de Ética en Investigación de la Clínica Avendaño (código: 017-2021-CIEI), y el Comité de Ética del HRHD mediante oficio N°-261-2021 GRA/GRS/GR-HRHD/DC-OCSI, para su autorización. Asimismo, fue registrado en la plataforma PRISA del Instituto Nacional de Salud con código EI00000002465.

## RESULTADOS

Se contactó a 125 médicos y 173 profesionales no médicos, de los cuales 60 médicos y 69 profesionales no médicos aceptaron participar y respondieron correctamente (tasa de no respuesta de 52% y 60,1%, respectivamente). De los profesionales no médicos, 60 (87%) fueron enfermeros/as, 5 (7,2%) obstetras y 4 (5,8%) psicólogos/as. La proporción de mujeres fue mayor en los profesionales no médicos (100%) en comparación con los médicos (58,3%). El promedio de experiencia laboral fue de 24,5 años en los médicos y de 28,6 años en los no médicos. La proporción de médicos con experiencia previa en telesalud fue mayor a la de los profesionales no médicos (20% vs 1,5%); la cantidad de no médicos que trabajaban diariamente con el sistema de telesalud fue mayor (50% vs 89,9%) (Tabla 1).

**Tabla 1:** Características del personal de salud que realizó teleconsultas en el Hospital Regional Honorio Delgado de Arequipa (N=129)

Características		Médicos n=60 n (%)	No médicos n=69 n (%)	Valor de p
Sexo				
	Femenino	35 (58,3)	69 (100,0)	<0,001^a^
	Masculino	25 (41,7)	0 (0,0)	
Grupo etario				
	Jóvenes y adultos (18-59)	36 (60,0)	27 (39,1)	0,018^b^
	Adulto mayor (≥60)	24 (40,0)	42 (60,9)	
Años de experiencia profesional		24,5 (10,5)	28,55 (13,9)	
Meses de experiencia en telesalud		11,93 (4,6)	12,22 (4,5)	
Experiencia previa en telesalud				
	Sí	12 (20,0)	1 (1,5)	0,001^a^
	No	48 (80,0)	68 (98,5)	
Frecuencia de atención				
	Diariamente	30 (50,0)	62 (89,9)	<0,001^a^
	Semanalmente	27 (45,0)	7 (10,1)	
	Mensualmente	3 (5,0)	0 (0,0)	
Percepción del uso de la tecnología				
	Principiante	8 (13,3)	17 (24,6)	0,278^b^
	Intermedio	41 (68,3)	41 (59,4)	
	Avanzado	11 (18,4)	11 (16,0)	

a Prueba exacta de Fisher; ^b^ Chi cuadrado

Fueron contactados 1 912 pacientes, la tasa de no respuesta fue de 80,3% pero esto no alteró el tamaño muestral calculado (n=377). El 76,7% fueron mujeres y la media de edad fue de 33,8 y 35,5 años para las mujeres y hombres, respectivamente. La mayor cantidad de población tenía grado de instrucción secundaria. La mayoría contaba con Seguro Integral de Salud (82,2%) y procedía de la provincia de Arequipa (86,7%). La modalidad de teleorientación fue la más utilizada (83%). El analfabetismo digital (referido al nivel de desconocimiento de las nuevas tecnologías y la necesidad de apoyo en el uso de tecnología por parte de los pacientes) fue de 46,2% (Tabla 2). 

**Tabla 2:** Características de los pacientes atendidos por el servicio de telesalud del Hospital Regional Honorio Delgado de Arequipa por llamada telefónica (N=377)

a Otras provincias de la región de Arequipa distintas de la provincia de Arequipa;

b Enfermero/a, obstetra, psicólogo/a

### Satisfacción del personal de salud

La proporción de profesionales no médicos satisfechos y completamente satisfechos fue significativamente mayor a la de los médicos en los criterios de satisfacción evaluados. El 69,6% de los profesionales no médicos manifestó sentirse satisfecho y completamente satisfecho con el resultado de la cita por telesalud en comparación al 38,3% de médicos. Asimismo, el nivel de satisfacción con la plataforma "Teleatiendo" de los profesionales no médicos durante su última cita fue mayor que en los médicos (72,4% vs 18,3%). Lo mismo ocurrió con la percepción del nivel de satisfacción por parte de sus pacientes en profesionales no médicos y médicos (87% y 28,3% respectivamente) (Tabla 3). El resumen de los comentarios y sugerencias del PS se encuentra en el material suplementario, anexos 2 y 3.

**Tabla 3:** Nivel de satisfacción con el servicio de teleconsulta por parte del personal de salud del servicio de telesalud del Hospital Regional Honorio Delgado de Arequipa (N=129).

Preguntas	Muy de acuerdo	De acuerdo	Neutro	En desacuerdo	Muy en desacuerdo	Valor de p
¿El haber realizado la cita podría cambiar (mejorar) el pronóstico del paciente?						
Médicos	6 (10,0)	21 (35,0)	18 (30,0)	8 (13,3)	7 (11,7)	0,004^b^
No médicos^a^	3 (5,0)	32 (46,4)	10 (14,5)	7 (10,1)	1 (1,5)	
El proceso de toma de decisiones clínicas se logró satisfactoriamente							
Médicos	3 (5,0)	14 (23,3)	17 (28,3)	14 (23,3)	12 (20,0)	0,001^b^
No médicos^a^	10 (14,5)	30 (43,5)	20 (29,0)	7 (10,1)	2 (2,9)	
	Completamente satisfecho	Satisfecho	Neutro	Insatisfecho	Completamente insatisfecho	
¿Qué tan satisfecho se siente con el resultado de la cita?							
Médicos	3 (5,0)	20 (33,3)	13 (21,7)	17 (28,3)	7 (11,7)	<0,001^b^
No médicos^a^	16 (23,2)	32 (46,4)	17 (24,6)	4 (5,8)	0 (0,0)	
¿Qué tan satisfecho se sintió con la plataforma de talesalud durante su última cita?							
Médicos	2 (3,3)	9 (15,0)	18 (30,0)	20 (33,3)	11 (18,3)	<0,001^b^
No médicos^a^	15 (21,7)	35 (50,7)	12 (17,4)	5 (7,3)	2 (2,9)	
¿Cómo considera el grado de satisfacción del paciente durante su última cita?							
Médicos	5 (8,3)	12 (20,0)	13 (21,7)	19 (31,7)	11 (18,3)	<0,001^b^
No médicos^a^	26 (37,7)	34 (49,3)	7 (10,1)	2 (2,9)	0 (0,0)

a Enfermero/a, obstetra, psicólogo/a; ^b^ Chi cuadrado

### Satisfacción de los pacientes

El 77,7% de pacientes manifestó estar muy de acuerdo o de acuerdo con respecto a sentirse satisfechos con el servicio de telesalud. Siete de los trece criterios evaluados tuvieron niveles altos de satisfacción, entre el 79,8% ("Volvería a utilizar los servicios de telemedicina") y el 93,6% ("Pude oír claramente al personal de salud"). Mientras que, en los seis restantes, la satisfacción osciló entre el 60,2% ("Pude ver al PS como si nos estuviéramos conociendo en persona") y el 75% ("No necesité ayuda para utilizar el sistema") (Figura 1).

**Figura 1:** Nivel de satisfacción con el servicio de teleconsulta por parte de los pacientes atendidos por el servicio de telesalud del Hospital Regional Honorio Delgado de Arequipa (N=377)

De los pacientes satisfechos, el 76,5% fueron mujeres y el 83% fueron atendidos por modalidad de teleorientación. El servicio de pediatría presentó mayor grado de satisfacción en comparación a otras especialidades (87%), seguido de ginecología y obstetricia (79,1%). Se observó que "seguimiento" fue el motivo de cita para 54,3% de los pacientes satisfechos. Se encontró un nivel de satisfacción similar entre los pacientes atendidos por el personal médico (76,3%) y no médico (77,7%). De los motivos de cita, "consulta" tuvo la mayor proporción de no satisfechos en comparación a los satisfechos (22,2% vs 14%) (Tabla 4). El resumen de los comentarios y sugerencias de los pacientes se encuentra en el material suplementario, anexo 4.

**Tabla 4:** Características de los pacientes atendidos según nivel general de satisfacción con el servicio de telesalud del Hospital Regional Honorio Delgado de Arequipa (N=377).

a Media (desviación estándar); ^b^ Otras provincias de la región de Arequipa distintas de la provincia de Arequipa; ^c^ Enfermero/a, obstetra, psicólogo/a; ^d^ U de Mann-Whitney;

e Chi cuadrado; ^f^ Prueba exacta de Fisher

### Nivel de madurez del sistema de telesalud

De forma global, 32% de ítems se encontraron en nivel nulo, 40,8% en iniciado, 25,2% en avanzado, y 2% en listo. De las seis categorías evaluadas, los aspectos normativos (Normas y procedimientos para la provisión de servicios de Telemedicina) y la preparación organizacional (Bases que identifican temas a resolver antes de avanzar con los servicios de telemedicina) se encuentraban principalmente en nivel avanzado, mientras que en la categoría de conocimiento especializado (conocimiento adicional para que beneficia una implementación efectiva) predominó el nivel nulo (Figura 2). La evaluación completa se muestra en el material suplementario, anexo 5.

**Figura 2:** . Nivel de madurez del servicio de telesalud del HRHD por categorías según la herramienta de medición del nivel de madurez de las instituciones de salud para implementar servicios de telemedicina de la OPS.

## DISCUSIÓN

La satisfacción del PS no médico fue mayor a la del personal médico con respecto a la toma de decisiones clínicas a través de la teleconsulta, con el resultado de la cita, con la plataforma "Teleatiendo" y con la percepción de la satisfacción de sus pacientes atendidos. En contraste, una revisión sistemática reporta que los médicos tienen altos niveles de aceptabilidad, viabilidad y adecuación con el sistema de telesalud y que sus pacientes parecían muy satisfechos con las atenciones de telesalud ^8^; sin embargo, estos estudios se caracterizaron por el uso de teleconferencias por video. La baja satisfacción de los médicos en nuestro estudio puede deberse a que estos necesitan del examen físico para lograr una evaluación adecuada, lo cual no es posible al realizar las consultas mediante llamadas telefónicas. En ese sentido, la mayor satisfacción del PS no médico se explicaría por su labor asistencial, la cual se centra en las modalidades de teleorientación y telemonitoreo, en las que la llamada telefónica es adecuada o suficiente. Esta situación mejoraría al realizarse atenciones de telesalud a través de videollamada pues se contaría al menos con la observación (examen físico) como base para establecer un mejor juicio clínico.

La satisfacción global de los pacientes fue menor en comparación con estudios en Latinoamérica, que reportaron mayor satisfacción (96% y 92,2%) ^15^^-^^17^, así como en otros continentes ^8^, esto podría explicarse por ser la única forma de cita disponible, diferencias del sistema de salud y/o mayor cobertura de las expectativas de atención en salud de los pacientes en otros países. La mayor satisfacción observada en pacientes atendidos por pediatría, seguido de ginecología y obstetricia, podría deberse a las facilidades que otorga la telesalud para estas especialidades, sin embargo, el deseo de futuras visitas por telesalud se vería significativamente afectado por su experiencia digital, la percepción de la necesidad de contacto físico, el tiempo ahorrado en viajes y el acceso a proveedores de salud ^18^. Hubo mayor nivel de insatisfacción en pacientes atendidos en cirugía, que podría deberse a la falta de atención por videollamadas ^19^. 

La modalidad en "Teleatiendo" más usada fue la teleorientación (83,0%), lo que es similar a lo reportado por la red de telesalud del Perú ^20^; pero diferente a lo reportado en España, donde la modalidad más usada fue telemonitoreo (65,9%) ^21^, principalmente para pacientes crónicos, en quienes la telesalud parece ser más efectiva y compatible ^22^. Se encontró que la "modalidad en Teleatiendo" difiere con el "motivo de consulta" reportado; esto puede deberse a la transformación de la cita, desde que es solicitada hasta la atención final requerida por el paciente y registrada en el sistema de "Teleatiendo". 

La satisfacción con el motivo de consulta de "seguimiento", (82,0%), fue similar a lo reportado en Portugal y España, probablemente debido a mayor frecuencia de citas y mejor relación con el PS ^21^^,^^23^. Así mismo, parece que los pacientes adultos mayores muestran menos interés en el seguimiento por telesalud ^21^, por lo que corresponde crear estrategias para incrementar la satisfacción de esta población y mejorar la atención de acuerdo a sus necesidades. El nivel de insatisfacción de pacientes con primera cita (22,3%) y citas anteriores (22,1%) fue alta, a diferencia del nivel de insatisfacción menor al 7% en la primera cita reportado en un estudio previo ^23^, esto se debería a las expectativas no cubiertas de los pacientes con respecto a la necesidad de un examinador en la primera cita. Estudios previos sugieren modelos que permitan una primera cita presencial y posteriores atenciones vía telesalud ^22^, propuesta que deberá ser examinada a futuro.

La telesalud - y la salud digital - pueden superar límites geográficos y contribuir a una mejor calidad de la atención, acelerando el progreso hacia la cobertura universal en salud ^24^. Pero esto sólo se logrará al acortar las brechas entre los servicios digitales, proveedores de salud y pacientes. Algunas de las brechas digitales son: el analfabetismo digital, limitado acceso a internet de ancho de banda amplio (alta velocidad), asequibilidad de dispositivos (celulares inteligentes, tabletas y computadoras), y difícil captación de pacientes de zonas rurales ^25^. Algunas de estas brechas se visibilizaron en este estudio. La cantidad de pacientes que requieren apoyo de un familiar para la cita por telesalud (más del 28%) es mayor a otros estudios (18% en España y 20% en Corea) ^9^^,^^26^. Se evidenció también un limitado aprovechamiento de la tecnología e internet, debido a que solo un 1,9% saca cita por internet y el 54,7% de pacientes saca cita por teléfono, la cual demora en concretarse debido a los tiempos de espera. Esto difiere de otros estudios internacionales, donde principalmente se saca citas por internet, lo cual presenta mayor nivel de satisfacción ^27^.

La satisfacción con el servicio de telesalud permite conocer si la atención médica se brinda de manera correcta y si cumple con las expectativas de los usuarios. Es importante que los tomadores de decisiones consideren la satisfacción de pacientes y personal de salud en la estandarización de planes de acción para la implementación, perfeccionamiento y seguimiento en telesalud ^28^.

Actualmente no existe una metodología estándar para evaluar el estado de madurez de un sistema de telesalud. La herramienta de evaluación de preparación de telesalud (TRA) ^29^ evalúa dominios similares a los considerados en la herramienta de medición del nivel de madurez de la OPS; el entorno digital y las operaciones normativas no están incluidos.

Los ítems de aspectos normativos y preparación organizacional se encontraron en estado avanzado o listo, ello indica que el servicio de telesalud se inició de manera parcial y de forma segura ^14^. Por otro lado, los ítems de "conocimiento especializado", "recursos humanos", "entorno digital" y "procesos", al tener un estado nulo o iniciado, reflejaron la necesidad de planificar acciones enfocadas, explicado en parte por la implementación apresurada de aplicaciones digitales en la atención de usuarios generada por COVID-19, y la falta de normativas y guías estandarizadas en el área ^4^^,^^30^. Expertos internacionales mencionan que los factores de éxito a considerar son; el desarrollo de marcos técnicos de acción de estándares internacionales, trabajadores de la salud capacitados y usuarios de salud alfabetizados digitalmente ^4^, estos dos últimos escasean en el sistema de telesalud del HRHD.

El presente estudio es el primero en evaluar la satisfacción de los usuarios y el nivel de madurez de un servicio de telesalud implementado en un hospital de Perú, a diferencia de otros estudios Latinoamericanos que estudiaron solo un servicio o especialidad. Con respecto a las, limitaciones, la principal fue que el instrumento de Glasser *et al*. no se encuentra validado; sin embargo, fue elegido debido a que ha sido utilizado en estudios previos para poder establecer comparaciones similares, además fue uno de los que mejor desempeño mostró comparado con otros instrumentos utilizados en investigaciones previas, los cuales contaban con preguntas enfocadas en los pacientes y no en el personal de salud. Los instrumentos utilizados estuvieron enfocados en consultas por videollamada, pero en el HRHD las consultas fueron por llamada telefónica, por lo que se adecuaron algunas preguntas sin que esto cambiara de forma sustancial el propósito de las preguntas. Adicionalmente se tuvo una alta tasa de no respuesta durante el muestreo. Se investigó un solo hospital, por lo que su reproductibilidad externa es baja, sin embargo, brinda una aproximación realista a lo que estaría sucediendo en otros centros de la Red Nacional de Telesalud de Perú.

En conclusión, el grado de satisfacción del personal de salud no médico fue moderadamente alta, contrastada con la baja satisfacción del personal médico. Los pacientes mostraron un grado de satisfacción con la telesalud moderadamente alto, aunque barreras como el analfabetismo digital y, limitaciones tecnológicas aún son barreras para la telesalud en el Perú. El nivel de madurez de implementación de la telesalud en el HRHD se encuentra más orientado hacia el nivel iniciado, por lo que será necesario hacer mejoras incrementales. Se recomienda que futuras investigaciones estudien servicios de telesalud en otros contextos a nivel nacional. Además, se recomienda realizar estudios con diseños que profundicen en el estudio de la satisfacción como tipo factores asociados y estudios cualitativos. Serán necesarios más estudios post-pandemia para evaluar la evolución de la satisfacción de los pacientes y el PS, así como el estado de la implementación o madurez del sistema de telesalud.
